# Orbital granular cell tumor involving the superior rectus muscle: a case report

**DOI:** 10.3389/fonc.2024.1456960

**Published:** 2024-11-07

**Authors:** Pei Wang, Zijian Han, Li Peng, Xiuhong Li, Hongfeng Yuan

**Affiliations:** ^1^ Department of Orbital Surgery, Chongqing Aier Eye Hospital, Chongqing, China; ^2^ Aier School Ophthalmology, Central South University, Changsha, Hunan, China; ^3^ Department of Pathology, Chongqing Emergency Medical Center, Chongqing, China

**Keywords:** orbital tumor, granular cell tumor, superior rectus muscle, immunohistochemistry, clinicopathological characteristics

## Abstract

**Objective:**

The aim of this case report is to assess the clinicopathological characteristics and differential diagnosis of orbital granular cell tumor (GCT).

**Methods:**

Clinical and imaging data of a rare case of orbital GCT involving the superior rectus muscle were collected. Its clinical characteristics, imaging, and histopathological features were observed.

**Results:**

A 36-year-old female patient presented with a 2-year history of left eye proptosis. Magnetic resonance imaging (MRI) enhancement suggested a space-occupying lesion in the left superior rectus muscle region. On T1-weighted and T2-weighted MRI, the tumor was isointense to gray matter and significantly enhanced on the enhanced scan. Microscopic examination revealed that most tumor cells exhibited diffuse growth with unclear boundaries, and some cells were arranged in small nests. The tumor cells were large, with abundant, coarse eosinophilic granules in the cytoplasm. Occasional cells contained larger round eosinophilic droplets in the cytoplasm. Focal areas showed foamy cells, small and central round or oval nuclei with occasional nuclear enlargement and mild atypia, inconspicuous nucleoli, rare mitoses, and low proliferative activity. Immunohistochemistry results were Vimentin (+), S-100 (+), CD68 (+), Ki67 (2%+), Inhibin-a (−), CK (−), SMA (−), and Desmin (−). The pathological examination of a specimen harvested from the mass corresponded to a GCT.

**Conclusion:**

Orbital GCT is rare and should be considered in the differential diagnosis of orbital tumors. It is essential to distinguish it from thyroid-associated ophthalmopathy, inflammatory pseudotumor, and myohemangioma. Definitive diagnosis requires a comprehensive analysis of clinical, histopathological, and immunohistochemical findings. Surgical excision is the primary treatment for orbital GCTs. For patients with incomplete tumor resection, close follow-up is necessary. Proton beam radiation therapy can be considered to prevent recurrence or metastasis if needed.

## Introduction

Granular cell tumor (GCT), also known as granular cell myoblastoma, is a rare benign tumor with neuroectodermal differentiation and is now considered a neurogenic tumor derived from Schwann cells (1). In the 2013 (fourth edition) World Health Organization classification of soft tissue tumors, it was classified as a nerve sheath tumor (2). GCTs are mostly benign, with malignancy accounting for only 0.5%–2% but with a mortality rate of 60%, and some benign GCTs can become malignant (3). GCTs commonly occur in females and can appear anywhere in the body, with 70% of cases in the head and neck, the tongue being the most common site, and GCTs in the orbit are rare (4–9). Clinical manifestations vary depending on the tumor’s location. In the eyelid, it can cause ptosis, whereas, in the retrobulbar area, it can lead to proptosis, motility restriction, localized conjunctival congestion, diplopia, and visual impairment (10). This article reports a rare case of orbital GCT involving the superior rectus muscle, with a discussion of its clinicopathological features, immunophenotype, and differential diagnosis based on the literature.

## Case report

**Figure 1:**
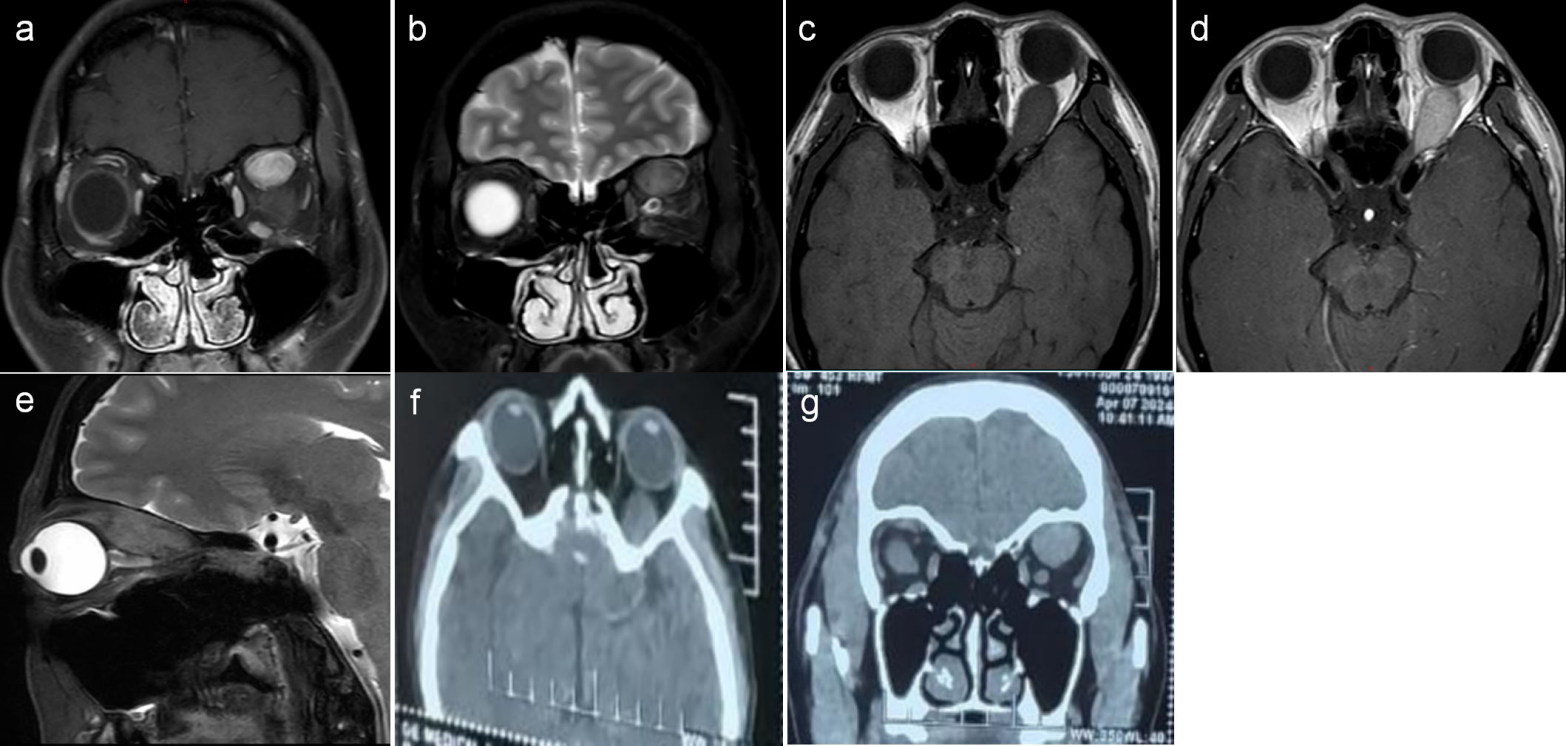
**(A–E)** Magnetic resonance imaging (MRI) showed a clear, oval mass in the left superior rectus muscle region, with intermediate T1 and slightly longer T2 signal intensity. The lesion was significantly enhanced on the scan, with small patchy weak enhancement areas visible inside. **(F, G)** Computed tomography (CT) scan showed a mass in the posterior cone of the left orbit, with an unclear boundary with the superior rectus muscle.

A 36-year-old female patient presented with a 2-year history of left eye proptosis. The patient had no history of hyperthyroidism, and preoperative thyroid function tests were normal. Systemic examination revealed no abnormalities, and both electrocardiogram and laboratory tests were unremarkable. The patient’s ophthalmic examination showed visual acuity of 0.12 for both eyes and best corrected visual acuity of 1.0 for both eyes. The visual acuity of both eyes was not affected, and no obvious abnormalities were observed in the anterior segment and fundus. Eye movement indicates limited upward and downward movement of the left eye, whereas normal movement of the right eye in all directions. Hertel’s exophthalmos examination showed exophthalmos of 12 mm in the right eye and 16 mm in the left eye, which revealed a 4-mm proptosis in the left eye. Magnetic resonance imaging (MRI) enhancement showed a clear, oval mass in the left superior rectus muscle region. On T1-weighted and T2-weighted MRI, the tumor was isointense to gray matter, and the left eyeball protrusion and left optic nerve compression were observed, with a significantly enhanced lesion on the enhanced scan and small patchy weak enhancement areas visible inside (**Figures 1A–E**). Imaging examination suggested inflammatory pseudotumors or neurogenic tumors. Computed tomography (CT) scan indicated a mass in the posterior cone of the left orbit, with an unclear boundary with the superior rectus muscle (**Figures 1F, G**). The patient underwent a left orbital mass biopsy, and the surgery is performed under general anesthesia, with a skin incision of about 2 cm below the left eyebrow arch. An electric knife is used to cut through the skin, subcutaneous tissue, and orbital periosteum along the eyebrow arch; open the orbital septum; and bluntly separate into the orbit to expose the tumor. After separating the surrounding tissue revealing a yellowish-white, hard tumor with poorly defined boundaries, tightly adhered to the superior rectus and levator palpebrae superioris muscles, and extending to the orbital apex. Complete excision was not feasible due to firm adhesion to the muscles and surrounding tissues, so a partial resection was conducted. The tumor was partially removed in blocks and was sent for pathological examination.

**Figure 2:**
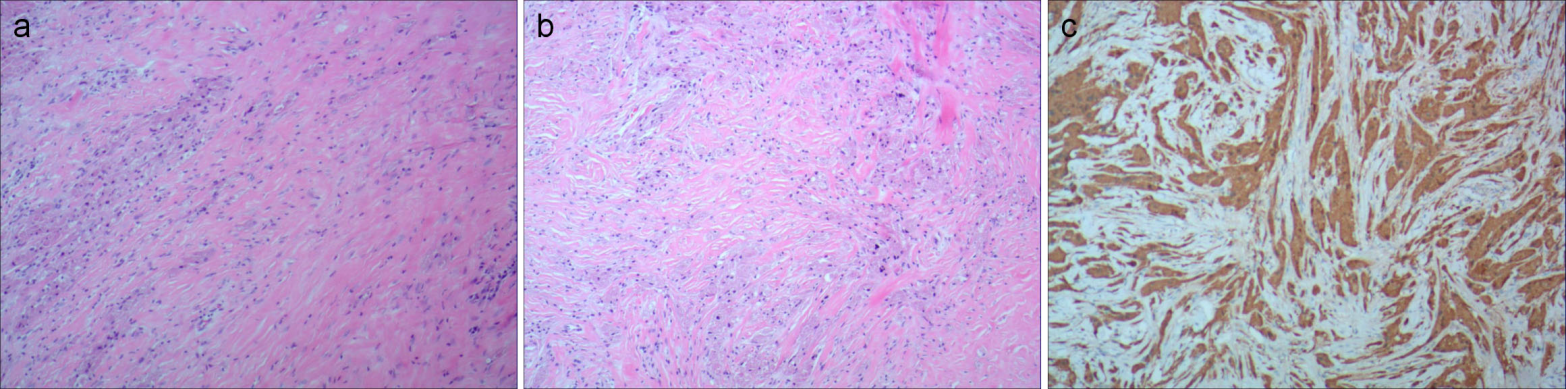
**(A, B)** Pathological photos showing tumor cells have abundant cytoplasm, eosinophilic, granular, small, and deeply stained nuclei (hematoxylin and eosin). **(C)** Immunohistochemical staining for S-100, Vimentin, and CD68 is positive.

Pathological examination revealed diffuse growth of tumor cells with unclear borders, some arranged in small nests. The tumor cells were large with abundant, coarse eosinophilic granules in the cytoplasm, occasional large round eosinophilic droplets, foamy cells, small central round or oval nuclei, occasional nuclear enlargement, mild atypia, inconspicuous nucleoli, rare mitoses, and low proliferative activity (**Figures 2A, B**). Immunohistochemistry results were Vimentin (+), S-100 (+), CD68 (+), Ki67 (2%+), Inhibin-α (−), CK (−), SMA (−), and Desmin (-), confirming the diagnosis of a GCT (**Figure 2C**).

## Discussion

We report a rare case of orbital GCT involving the superior rectus muscle. Orbital GCTs are extremely rare, with only about 60 cases reported (11). Our patient was a 36-year-old female, whereas the average age of onset for orbital GCTs is 44 years (11). In reported cases, 70% involve the extraocular muscles, possibly due to the tumor’s neural origin and development associated with small- to medium-sized nerves supplying these muscles (12). S-100 positivity in immunohistochemistry suggests a neurogenic origin (6). The inferior rectus muscle is most commonly affected, followed by the medial rectus, with superior rectus involvement being very rare (12). As most tumors involve the extraocular muscles, diplopia is the most common ocular symptom (6, 10). However, our patient’s tumor mainly affected the superior rectus muscle, which is relatively rare. The main manifestation of eye movement is limited upward and downward rotation, and the patient did not have obvious diplopia before surgery, with eyeball protrusion being the primary complaint.

Symptoms of GCTs depend on the location, most occur in the inferior orbit, causing symptoms like decreased vision and pain (13). Orbital GCTs rarely involve the optic nerve and parasympathetic ciliary ganglion, and tumors in the superior orbit often cause pain, perhaps due to the tumors disturbing the parasympathetic ciliary ganglion, causing a tonic pupil with impairment in accommodation and contributing to pain in the eye (14). In our case, the tumor was located above the orbit but did not cause these symptoms. Orbital GCT patients typically present with proptosis, diplopia, or blurred vision, and imaging diagnosis lacks specificity, often leading to misdiagnosis based solely on CT and MRI.

Most orbital GCTs manifest as a well-defined, round, or oval mass on imaging studies (15). No calcification or bony remodeling is observed because of its predominant intramuscular location (13). CT without contrast reveals an isointense or slightly hyperdense tumor relative to the brain tissue. On T1-weighted MRI, the tumor is isointense to gray matter and hypointense on T2. With contrast, there is a slight to strong enhancement (9, 13). In this case, MRI showed a clear elliptical mass in the upper rectus muscle walking area, with clear boundaries and regular morphology. On T1-weighted and T2-weighted MRI, the tumor is isointense to gray matter. The enhanced scan showed significant enhancement of the lesion, with small patchy weak enhancement areas visible inside. The tumor in our case largely exhibits typical MRI features of granulosa cell tumors.

Previous articles on orbital GCT case reports have found that, through imaging examinations, orbital GCT is often misdiagnosed as inflammatory pseudotumor, thyroid-associated ophthalmopathy, and myohemangioma (16, 17). Although most inflammatory pseudotumors of the orbit show slightly longer T1 and slightly shorter T2 signal shadows on plain scan, most of them show uniform and significantly enhanced shadows. Because of the presence of flowing blood, blood vessels, and fibrous tissue within the tumor, myohemangioma shows high-signal shadows with bead-like, strip-like, and small plaques in T1W1 but low-signal shadows with dot-like and worm-like features in T2W1. Although thyroid-associated ophthalmopathy manifests as thickening of the extraocular muscles primarily in the muscular abdomen, almost all thickened ocular muscles show slightly high-signal intensity on T2W1, due to edema being the cause of most extraocular muscle thickening. CT scans of orbital GCTs often present as soft tissue density shadows, appearing as diffuse or elliptical masses with equal density relative to brain tissue, without calcification, and adjacent bone masses without involvement. GCTs located within or near the extraocular muscles on CT scans are similar to thyroid-related eye diseases, but MRI may, sometimes, reveal the boundary between the tumor and the rectus muscle. When MRI shows similar features, a pathological examination can be performed to confirm the findings. In addition, for extraocular muscle hypertrophy without obvious causes, some patients may receive treatment for thyroid-related eye diseases. The possibility of orbital tumors is considered only if there is no effect. When the initial diagnosis of thyroid-associated ophthalmopathy and treatment for inflammatory pseudotumors have no effect, a differential diagnosis should be made with granulosa cell tumors.

GCTs of the orbit are rare, and, although imaging can be helpful for assessing a differential diagnosis, a biopsy is required for a definitive diagnosis (14). The diagnosis of GCT relies primarily on pathological examination, revealing round or polygonal eosinophilic granular cells in nests, sheets, or broad bands, separated by fibrous connective tissue. Tumor cells are uniform, with abundant eosinophilic cytoplasm containing Periodic Acid-Schiff (PAS)-positive granules (5, 18). Immunohistochemistry shows positivity for *S-100 protein, NSE, myelin basic protein, CD68, calretinin, Inhibin-α*, and *TFE3*. (19). The tumor has no obvious capsule, so tumor cells can invade surrounding soft tissue. Features of malignant GCTs include increased cellularity, pleomorphism, vesicular nuclei, prominent nucleoli, necrosis, increased mitotic activity, and a high Ki-67 index (10, 11, 20). Previously published articles involving GCT of the orbit found 60 reported cases, in which only 6 cases involved the superior rectus muscle (14). A diagnosis of GCT is made on the basis of a histopathologic examination that shows characteristically abundant eosinophilic granular cytoplasm and finely granular cytoplasm (19, 21). These fine granules are periodic acid–Schiff–positive and diastase-resistant and can occasionally aggregate in globules surrounded by a halo (called Milian bodies) (20). S100 confirm the neurogenic nature of GCTs. Lysosomes can be highlighted by the CD68 stain (10, 14, 22). Immunohistochemical and electron microscopic studies have recently indicated that GCT represents a neural Schwann cell–related neoplasm (5, 23). Compared with other reported similar cases, our case also exhibited typical pathological features common to GCTs with immunohistochemistry showing positivity for markers such as Vimentin, S-100, and CD68 that could help in subsequent diagnosis.

Surgical excision is the standard treatment for orbital GCTs (6, 10). However, because of the frequent involvement of extraocular muscles, diplopia often persists even after complete excision (6, 8, 24). When tumors adhere to surrounding tissues or are located in the posterior orbit, complete excision is challenging. Although recurrence is possible, aggressive behavior, including local invasion and metastasis, is rare (6, 10, 14). In our case, we were unable to completely remove the tumor because it was located deep into the orbital apex and firmly adhered to the muscles and surrounding orbital soft tissue. In order to minimize the impact on the patient’s vision, we chose partial resection. Literature indicates a relatively low recurrence rate (10%) for partially resected cases (6). Close follow-up is necessary for patients with incomplete excision, and proton beam radiation therapy (PBRT) can be considered to prevent recurrence or metastasis if needed (4, 6).

In this case, the Ki-67 index value of 2% strongly suggests that the present GCT was a benign GCT (24). Recent reports have shown that recurrences after surgery occur in approximately 10% of patients and depend largely on the tumor’s location (14). A tumor in the posterior orbit may be harder to reach by the surgeon and more difficult to be completely removed, with a higher likelihood of recurrence than if it were in the lateral orbit (14). Regular follow-up is required after surgery, during which MRI examinations are performed regularly. Eye examinations include monitoring exophthalmos and extraocular muscle motility and the degree of diplopia to determine if the tumor has further invaded the muscles. For tumors that have not been completely resected, it is recommended to have a MRI review every 6 months to detect the size of the lesion and observe the overall condition. Once tumor recurrence is detected, ultrasound examination can be performed, and systemic computed tomographic images can be performed if necessary to determine whether the tumor has systemic metastasis. Early recurrence can be considered with local radiotherapy. If radiotherapy is not sensitive, then surgical resection may ultimately be performed.

Barrantes et al. reported significant tumor regression with PBRT in cases where surgical excision was incomplete, highlighting its utility in managing benign orbital GCTs (14). Especially in cases of incomplete tumor removal, PBRT can alleviate symptoms, reduce residual tumor size, and have acceptable toxicity (14). Radiation therapy for benign orbital tumors has been considered the last resort for treatment (25–29). Its use has been controversial due to the deposition of dose beyond the target/tumor and, therefore, potential for the development of late toxicities (30). There are reports showing that benign GCT has relative radiation resistance to conventional radiation therapy (17). Compared with traditional radiation therapy and other types of focused radiation therapy such as intensity modulated radiation therapy and gamma knife radiation surgery, PBRT allows for providing sufficiently high and uniform doses to tumors without irradiating healthy tissues outside the target (31). Proton beams can be transmitted to depths of 1 mm to 32 cm with minimal dose upon entry, providing a maximum dose at the specified depth without exceeding the dose at that point. These unique features of PBRT are particularly relevant for treating complex and small anatomical structures, such as the eyeball and optic nerve. In recent years, the advancement of radiation delivery methods and the continuous understanding of the effects of radiation on the eyes have made PBRT a more widely used radiation method for orbital tumors (32). According to reports, due to direct and indirect DNA damage, the mechanism by which PBRT causes tumor shrinkage is more effective in actively dividing tumor cells (high replication index can be expressed through Ki-67 staining), thereby reducing cell replication and growth ability (33). It may affect the blood supply to small blood vessels, leading to ischemia, and may induce fibrosis, resulting in tumor cell shrinkage.

In summary, we report a rare case of orbital GCT involving the superior rectus muscle. Orbital GCTs are rare and should be considered in the differential diagnosis of orbital tumors. Attention should be paid to distinguishing them from thyroid-associated ophthalmopathy, inflammatory pseudotumors, intramuscular hemangiomas, etc. The diagnosis should be combined with clinical, histopathological, and immunohistochemical information for comprehensive analysis. Surgical resection is the main treatment for orbital GCTs. For patients whose tumors have not been completely removed by surgery, close follow-up should be conducted to prevent recurrence or metastasis. PBRT may also be considered if necessary.

## Data Availability

The original contributions presented in the study are included in the article/supplementary material. Further inquiries can be directed to the corresponding author.
